# Murine Hepatitis Virus, a Biosafety Level 2 Model for SARS-CoV-2, Can Remain Viable on Meat and Meat Packaging Materials for at Least 48 Hours

**DOI:** 10.1128/spectrum.01862-22

**Published:** 2022-09-07

**Authors:** Austin B. Featherstone, Amanda Claire Brown, Sapna Chitlapilly Dass

**Affiliations:** a Department of Animal Science, Texas A&M University, College Station, Texas, USA; Health Canada

**Keywords:** MHV, meat, SARS-CoV-2, packaging material

## Abstract

In 2020 and 2021, many meat processing plants faced temporary closures due to outbreaks of COVID-19 cases among the workers. There are several factors that could potentially contribute to the increased numbers of COVID-19 cases in meat processing plants: the survival of viable SARS-CoV-2 on meat and meat packaging materials, difficulties in maintaining workplace physical distancing, personal hygiene, and crowded living and transportation conditions. In this study, we used murine hepatitis virus (MHV) as a biosafety level 2 (BSL2) surrogate for SARS-CoV-2 to determine viral survival on the surface of meat, namely, stew-cut beef and ground beef, and commonly used meat packaging materials, such as plastic wrap, meat-absorbent material, and Styrofoam. From our studies, we observed the infectivity of MHV inoculated on ground beef and stew-cut beef for 48 h and saw no significant loss in infectivity for MHV from 0 to 6 h postinoculation (hpi) (unpaired *t* test). However, beginning at 9 hpi, viral infectivity steadily decreased, resulting in a 1.12-log reduction for ground beef and a 0.46-log reduction for stew-cut beef by 48 hpi. We also observed a significant persistence of MHV on meat packaging materials, with Styrofoam supporting the highest viability (3.25 × 10^3^ ± 9.57 × 10^2^ PFU/mL, a 0.91-log reduction after 48 hpi), followed by meat-absorbent material (75 ± 50 PFU/mL, a 1.10-log reduction after 48 hpi), and lastly, plastic wrap (no detectable PFU after 3 hpi, a 3.12-log reduction). Despite a notable reduction in infectivity, the virus was able to survive and remain infectious for up to 48 h at 7°C on four of the five test surfaces. Our results provide evidence that coronaviruses, such as SARS-CoV-2, could potentially survive on meat, meat-absorbent materials. and Styrofoam for up to 2 days, and potentially longer.

**IMPORTANCE** The meat industry has been faced with astronomical challenges with the rampant spread of COVID-19 among meat processing plant workers. This has resulted in meat processing and packaging plant closures, creating bottlenecks everywhere in the chain, from farms to consumers, subsequently leading to much smaller production outputs and higher prices for all parties involved. This study tested the viability of meat and meat packaging materials as potential reservoirs for SARS-CoV-2, allowing the virus to survive and potentially spread among the workers. We used murine hepatitis virus (MHV) as a biosafety level 2 (BSL2) surrogate for SARS-CoV-2. Our results suggest that ground beef, stew-cut beef, meat-absorbent material, and Styrofoam can harbor coronavirus particles, which can remain viable for at least 48 h. Furthermore, our study provides evidence that the environmental and physical conditions within meat processing facilities can facilitate the survival of viable virus.

## INTRODUCTION

Murine hepatitis virus (MHV) and severe acute respiratory syndrome coronavirus-2 (SARS-CoV-2) are both positive-sense RNA coronaviruses that can infect numerous mammalian species and can cause lethal disease (1, 2). At the start of the SARS-CoV-2 pandemic, there was no information on whether SARS-CoV-2 could be transmitted by food. SARS-CoV-2 is known to primarily infect alveolar epithelial cells and capillary endothelial cells by binding to the angiotensin-converting enzyme 2 (ACE2) receptor (1–3). However, alternative receptors have recently been discovered which SARS-CoV-2 could use to spread infection throughout the body (4). Alternative receptors for SARS-CoV-1 and SARS-CoV-2 include the C-type lectins: dendritic cell-specific intercellular adhesion molecule-3-grabbing nonintegrin (DC-SIGN) and liver/lymph node-specific intercellular adhesion molecule-3-grabbing integrin (L-SIGN) (5, 6). Other suggested receptors for SARS-CoV-2 include T-cell immunoglobulin and mucin domain 1 (TIM1), anexelekto (AXL) receptor tyrosine kinase, cluster of differentiation 147 (CD147), neuropilin 1 (NRP1), and sodium-dependent neutral amino acid transporter (B0AT1) (7–12), thus suggesting that body cells, including those found in human food, could harbor SARS-CoV-2.

Working with SARS-CoV-2 requires special training and biosafety level 3 (BSL3) laboratory conditions; therefore, there are significant challenges to studying SARS-CoV-2. Numerous studies have suggested that MHV is a good surrogate for studying SARS-CoV-2 due to their genetic similarity and comparable responses/levels of inactivation to antivirals and chemical disinfectants (13–15).

MHV and SARS-CoV-2 virions are stable at colder temperatures for several days on meat and poultry, as well as on surfaces that are commonly found in meat processing facilities (i.e., stainless steel, PVC, and ceramic tile) (16–19), suggesting that these plants have a potentially high risk of harboring and transmitting SARS-CoV-2 (19). At the start of the pandemic, many meat processing plants were forced to temporarily close due to surges in COVID-19 cases (20–22). Work from our group and others has identified many environmental factors present in meat processing plants which are responsible for the spikes in SARS-CoV-2 cases, including the close proximity of workers to one another, high levels of air circulation allowing the virus to spread throughout the facility, shared contact surfaces that multiple workers use, common transportation methods used by workers to travel to and from work, and plant worker housing where multiple staff often live together in potentially overcrowded conditions (20–22).

To determine whether meat and meat packaging materials, routinely found in meat processing facilities, could be a reservoir for SARS-CoV-2—allowing the virus to survive and potentially spread among the workers—we used MHV as a SARS-CoV-2 surrogate. To test viral survival, MHV was applied to two meat products (ground beef and stew-cut beef) and to three meat packaging materials (plastic wrap, meat-absorbent material, and Styrofoam) and incubated under the environmental conditions found within meat processing plants (7°C) for 48 h, in order to replicate the environmental conditions found within meat processing plants. reverse transcriptase quantitative PCR (RT-qPCR) and plaque assays were used to assess the presence and viability of the virus particles during incubation on the test surfaces.

## RESULTS

### MHV was able to remain viable and infectious on multiple surfaces for over 48 h.

**FIG 1:**
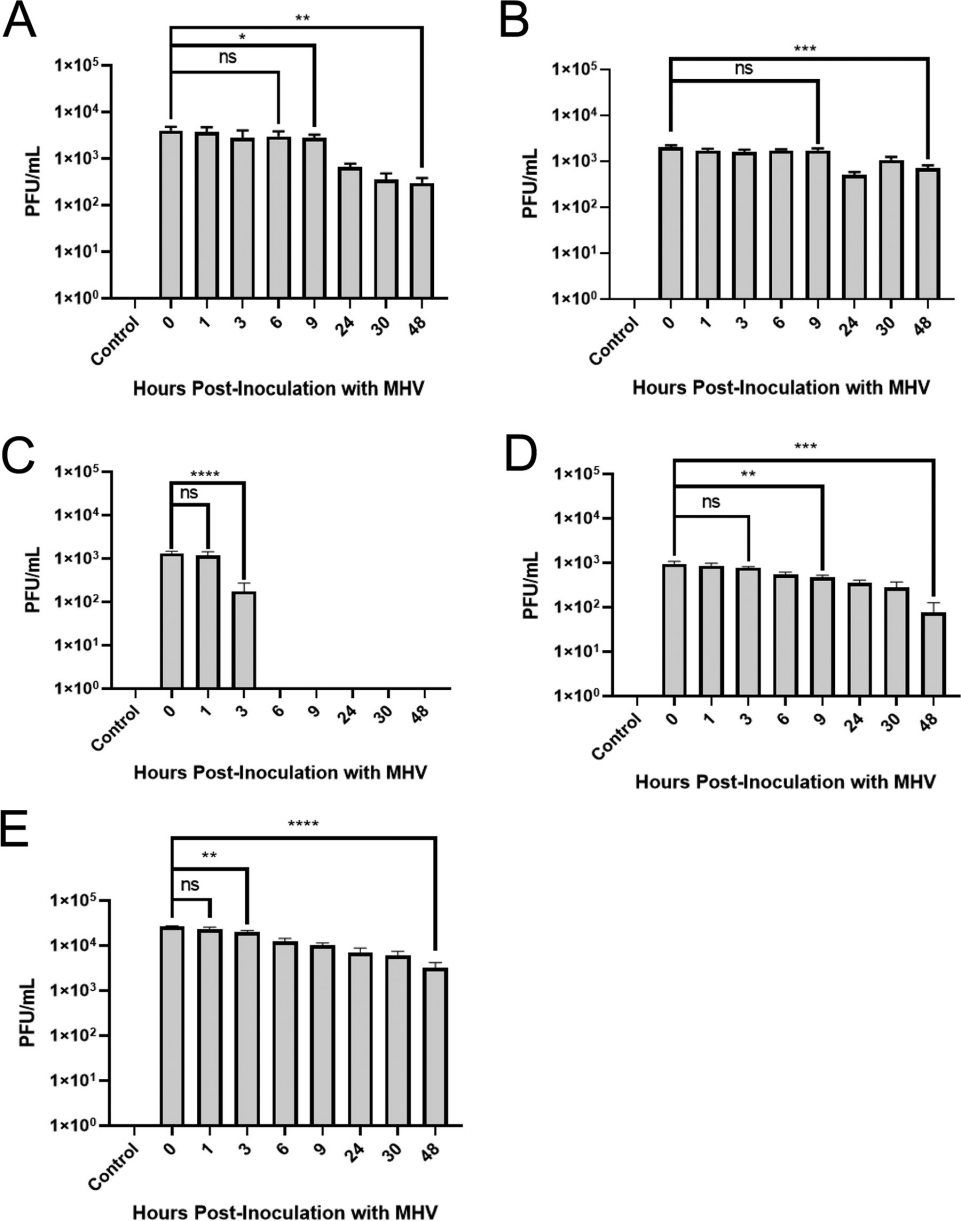
(A to E) Plaque assay analysis of MHV collected from (A) ground beef, (B) stew-cut beef, (C) plastic wrap, (D) meat-absorbent material, and (E) Styrofoam postincubation. Each sample was filtered through a 0.2-μm syringe filter and plated onto L2 cells in duplicate. Results in this figure are the mean values and standard deviations (error bars) from two independent experiments with technical duplicates for each sample in each experiment giving four data points per sample. Statistical significance was analyzed by unpaired *t* test. *, *P < *0.05; **, *P < *0.01; ***, *P < *0.001; ****, *P < *0.0001.

We wanted to determine whether MHV could remain infectious for up to 48 h postinoculation (hpi) on meat and meat packaging materials. MHV was inoculated onto ground beef (Fig. 1A), stew-cut beef (Fig. 1B), plastic wrap (Fig. 1C), meat-absorbent materials (Fig. 1D), and Styrofoam (Fig. 1E) and was collected over a span of 2 days at 0, 1, 3, 6, 9, 24, 30, and 48 hpi.

In terms of viral viability, there was an overall 1.12-log reduction in PFU/mL from 0 hpi to 48 hpi when incubated on ground beef (Fig. 1A). Specifically, the average PFU/mL for MHV on ground beef from 1 to 6 hpi was 3.38 × 10^3^ (± 9.62 × 10^2^), compared with 4.0 × 10^3^ (± 8.16 × 10^2^) at 0 hpi, and there was no statistically significant difference between these time points (Fig. 1A). At 9 hpi, there was a significant reduction in viral infectivity, where the PFU/mL dropped to 2.75 × 10^3^ (± 5.00 × 10^2^), representing a 0.16-log reduction in infectivity compared to that at 0 hpi. There was a significant decrease in infectious virus particles following further incubation on ground beef. PFU/mL dropped to 6.50 × 10^2^ (± 1.29 × 10^2^) at 24 hpi, 3.50 × 10^2^ (± 1.29 × 10^2^) at 30 hpi, and 3.00 × 10^2^ (± 81.60) at 48 hpi.

MHV was found to survive better on stew-cut beef than ground beef, where we observed a 0.46-fold reduction in PFU/mL from 0 hpi to 48 hpi, compared to a 1.12-log reduction for ground beef. The average PFU/mL for the stew-cut beef from 1 to 9 hpi was 1.75 × 10^3^ (± 1.74 × 10^2^) compared with 2.03 × 10^3^ (± 2.06 × 10^2^) at 0 hpi, suggesting no significant difference between these time points (Fig. 1B). As previously seen with ground beef, after 9 hpi there was a significant reduction in infectious virus collected at 24, 30, and 48 hpi. The average PFU/mL was 1.05 × 10^3^ (± 1.91 × 10^2^) at 30 hpi and 7.00 × 10^2^ (± 1.15 × 10^2^) at 48 hpi.

For all the meat packaging materials tested, plastic wrap was found to support the lowest level of viral persistence. The average PFU/mL for the plastic wrap from 0 to 1 hpi was 1.25 × 10^3^ (± 2.06 × 10^2^), and there was no significant difference between these two time points (Fig. 1C). At 3 hpi, the average PFU/mL on plastic wrap was 1.75 × 10^2^ (± 95.7), which was a 1.25-log reduction in infectivity compared to 0 hpi (Fig. 1C). From 6 to 48 hpi, we did not observe any infectious virus particles on the plastic wrap (Fig. 1C), suggesting that MHV is unable to survive on plastic wrap after 6 hpi (equivalent to a 3.12-log reduction).

Unlike plastic wrap, meat-absorbent material was found to be able to support viable virus over the entire 48-h period of the assay. The average PFU/mL for the meat-absorbent material from 0 to 3 hpi was 8.58 × 10^2^ (± 1.03 × 10^2^), and there was no significant difference between these time points (Fig. 1D). There was a significant reduction in the number of infectious virus particles at 6, 9, 24, 30, and 48 hpi (Fig. 1D); specifically, the amount of infectious virus was reduced to 5.50 × 10^2^ (± 57.7) at 6 hpi, 4.75 × 10^2^ (± 50.0) at 9 hpi, 3.50 × 10^2^ (± 57.7) at 24 hpi, 2.75 × 10^2^ (± 95.7) at 30 hpi, and 75 (± 50.0) at 48 hpi. Overall, this represented a 1.10-log reduction in viable virus particles from 0 to 48 hpi and was comparable to data observed with MHV incubated on ground beef (1.10-log reduction versus a 1.12-log reduction) (Fig. 1A and D).

MHV was found to remain viable on Styrofoam the longest of the three packaging materials tested. After the initial inoculation, the average PFU/mL from 0 to 1 hpi, was 2.46 × 10^4^ (± 1.92 × 10^3^), and there was no significant difference between these time points (Fig. 1E). However, from 1 hpi, there was a noticeable decrease in infectious virus particles (collected at 3, 6, 9, 24, 30, and 48 hpi), representing an overall 0.91-log reduction in infectious virus particles from 0 to 48 hpi. Specifically, the amount of infectious virus (in PFU/mL) was reduced to 2.0 × 10^4^ (± 1.63 × 10^3^) at 3 hpi, 1.23 × 10^4^ (± 2.06 × 10^3^) at 6 hpi, 1.03 × 10^4^ (± 1.26 × 10^3^) at 9 hpi, 7.00 × 10^3^ (± 1.83 × 10^3^) at 24 hpi, 6.00 × 10^3^ (± 1.41 × 10^3^) at 30 hpi, and 3.25 × 10^3^ (± 9.57 × 10^2^) at 48 hpi. MHV remained viable on Styrofoam over the 48 h at much higher numbers than on plastic wrap and meat-absorbent material (0.91-log reduction compared to no infectious virus at 48 hpi on plastic wrap [3.12-log reduction] and 1.10-log reduction on meat-absorbent material). Altogether, these results suggest that MHV can remain infectious (listed from highest viability) on stew-cut beef (0.46-log reduction), Styrofoam (0.91-log reduction), meat-absorbent material (1.1-log reduction), and ground beef (1.12-log reduction) for up to 48 hpi, but only on plastic wrap for up to 3 hpi (3.12-log reduction).

## DISCUSSION

In 2020, many meat processing plants globally had to close due to spikes in COVID-19 cases among workers (23). The National Cattlemen’s Beef Association predicts that the cattle industry will potentially experience a $13.6 billion loss due to the novel coronavirus, SARS-CoV-2, impacting ranchers across the country (24). In order to understand why SARS-CoV-2 had a huge spike in meat processing plants in 2020 and in 2021, we must better understand why meat processing plants are a great environment for SARS-CoV-2 to be able to survive and spread (20, 22, 25). From our current research, we have identified several factors that can contribute to the rise in COVID-19 cases in meat processing plants. SARS-CoV-2 is stable at lower temperatures, as meat processing plants are kept between 4 and 7°C (16, 26–28). With the cool temperatures in the meat processing plants, aerosol droplets containing virus will not evaporate as quickly compared to higher temperatures, potentially maintaining viral viability and persistence (29, 30). In addition, heating ventilation and air conditioning (HVAC) systems in the meat processing plants could easily transfer SARS-CoV-2 throughout the facility (31, 32). The virus can also easily be contracted by the workers because of the shared equipment and tools, along with close living, work, and travel arrangements to and from the meat processing plant (20, 22). However, one area of SARS-CoV-2 research that is still being explored is the potential for fomite-mediated transmission associated with food and food packaging surfaces in meat processing plants.

SARS-CoV-2 is considered a respiratory virus, infecting hosts by inhalation of aerosols or droplet nuclei containing virus—from person to person. However, our virus survival data suggest that other methods of infection could exist, such as from touching meat/packaging materials contaminated with SARS-CoV-2 and then transferring the virus to the nose/face via the hands. Alternatively, physical activities common in meat processing, such as spray chilling a carcass contaminated with SARS-CoV-2, could create virus-carrying aerosols which can spread throughout the facility. Therefore, we sought to identify whether SARS-CoV-2 could remain viable on different cuts of meat and on meat packaging materials in order to determine whether virus-contaminated meat and meat packaging materials could be an alternative route of infection for SARS-CoV-2.

In this study, we used MHV as a biosafety level 2 (BSL2) surrogate for SARS-CoV-2 to determine the survival of virus on the surfaces of two meat types, stew-cut beef and ground beef, and three types of typical meat packaging materials, plastic wrap, meat-absorbent material, and Styrofoam. These were used as a representation of different cuts of meat and packaging materials used in the meat processing plant. Other recent studies have investigated whether SARS-CoV-2 can infect pets, livestock, or wild animals and suggest that cattle and sheep have low susceptibility to SARS-CoV-2 infection, while swine and poultry have no identifiable susceptibility to SARS-CoV-2 (33–35). In addition, further studies have suggested that viral transfer from foods and packaging materials to gloves provides evidence that fomite transmission of SARS-CoV-2 is of minor importance (36, 37).

We used RT-qPCR targeting the M gene of MHV, which encodes the viral membrane protein. The SARS-CoV-2 M gene has been demonstrated as suitable for the identification of the presence and quantification of viral RNA (38). However, viral RNA detection does not demonstrate viability, even though RNA is typically labile and unlikely to remain present in the environment for long. Therefore, we performed plaque assays with our recovered samples to identify the amount of infectious virus in a sample, demonstrating viral viability. The RT-qPCR data are discussed further in the supplemental Results section.

Increased MHV viability after incubation on stew-cut beef compared to ground beef was identified by plaque assays (after 9 hpi, Table 1). When MHV was inoculated on ground beef, the physical characteristics of the meat (open texture, high fat content, and porosity) allowed the virus to be easily absorbed (39, 40). The presence of reactive proteins and enzymes in meat, for example, hemoproteins such as hemoglobin and myoglobin, which are both present in meat and tissues rich in blood, such as organs, could adversely affect viral viability (41, 42). During deoxygenation, nitric oxide (NO) reacts with oxygenated ferrous heme proteins present in myoglobin and hemoglobin, to form ferric heme and nitrate (NO_3_–) (41, 42). During this reaction, an excess of oxygen free radicals is released, and these can damage nearby viral RNA (vRNA) by binding to the vRNA, inhibiting viral translation and protein synthesis, and can also disrupt nearby cell membranes (43, 44). When the neighboring eukaryotic cells are damaged and lyse, harmful reactive enzymes such as nucleases and proteases are released, which can also degrade vRNA and alter the Env conformation of MHV. Alternatively, iron released from the disrupted red blood cells can bind to vRNA stem loops, thereby damaging the RNA and reducing the viability of the virus. Although we observed a significant decrease on both ground beef and stew-cut beef after 9 hpi, MHV was still infectious at 48 hpi on both meat cuts.

**TABLE 1:** Data from the plaque assay analysis of the recovered MHV viral particles from the different experimental conditions^*a*^

Sample	PFU/mL @ 0 hpi	PFU/mL @ 1 hpi (% change/fold change)	PFU/mL @ 3 hpi (% change/fold change)	PFU/mL @ 6 hpi (% change/fold change)	PFU/mL @ 9 hpi (% change/fold change)	PFU/mL @ 24 hpi (% change/fold change)	PFU/mL @ 30 hpi (% change/fold change)	PFU/mL @ 48 hpi (% change/fold change)
Ground beef	3,500 ± 707; 4,500 ± 707	(–14.3%, 1.17-fold); (–0.0%, 1.0-fold)	(–42.9%, 1.75-fold); (–22.2%, 1.29-fold)	(–28.6%, 1.4-fold); (–22.2%, 1.29-fold)	(–28.6%, 1.4-fold); (–33.3%, 1.5-fold)	(–84.3%, 6.36-fold); (–83.3%, 6-fold)	(–92.9%, 14-fold); (–90.0%, 10-fold).	(–91.4%, 11.67-fold); (–93.3%, 15-fold)
Stew-cut beef	2,150 ± 212; 1,900 ± 141	(–23.3%, 1.3-fold); (–5.3%, 1.06-fold)	(–25.6%, 1.34-fold); (–15.8%, 1.19-fold)	(–20.9%, 1.26-fold); (–10.5%, 1.12-fold)	(–18.6%, 1.23-fold); (–13.2%, 1.15-fold)	(–74.4%, 3.91-fold); (–76.3%, 4.22-fold)	(–48.8%, 1.95-fold); (–47.4%, 1.9-fold).	(–67.4%, 3.07-fold); (–63.2%, 2.7-fold)
Plastic wrap	1,300 ± 141; 1,350 ± 212	(–15.4%, 1.18-fold); (–7.4%, 1.08-fold)	(–80.8%, 5.2-fold); (–92.6%, 13.5-fold)	0; 0	0; 0	0; 0	0; 0	0; 0
Meat-absorbent material	1,050 ± 71; 850 ± 71	(–9.5%, 1.11-fold); (11.8%, 1.13-fold)	(–23.8%, 1.31-fold); (11.8%, 1.13-fold)	(–47.6%, 1.91-fold); (–35.3%, 1.55-fold)	(–57.1%, 2.3-fold); (–41.2%, 1.7-fold)	(–61.9%, 2.63-fold); (–64.7%, 2.83-fold)	(–81.0%, 5.25-fold); (–58.8%, 2.43-fold)	(–95.2%, 21-fold); (–88.2%, 8.5-fold)
Styrofoam	25,500 ± 707; 27,000 ± 1,410	(–13.7%, 1.16-fold); (–11.1%, 1.13-fold)	(–25.5%, 1.34-fold); (–22.2%, 1.29-fold)	(–56.9%, 2.32); (–50.0%, 2-fold)	(–62.7%, 2.68-fold); (–59.3%, 2.45-fold)	(–72.5%, 3.64-fold); (74.1%, 3.86-fold)	(–78.4%, 4.64-fold); (–75.9%, 4.15-fold)	(–88.2%, 8.5-fold); (–87.0%, 7.71-fold)

a PFU/mL numbers and the percentage and fold change from the initial inoculum (1.0 × 10^4^ PFU/mL).

The results from MHV exposed to meat packaging materials suggest that viral viability is significantly inhibited after 3 hpi on meat-absorbent materials and Styrofoam and after 1 hpi on plastic wrap (Fig. 1). The temperature of the meat processing facility is 4 to 7°C, and these temperatures are ideal for coronavirus virions to be stable and remain infectious (16). In addition, the cool temperatures mean liquid droplets holding virus will not evaporate as quickly as in an environment above 20°C, allowing increased viral viability (29, 30). When considering why the virus was able to survive on the meat-absorbent materials and on the Styrofoam longer than on the plastic wrap, there are several potential reasons. One observation was that when MHV was inoculated onto plastic wrap, the inoculum almost immediately spread out across the entire surface due to the adhesive forces of the plastic. This is a dynamic process known as static wetting, which causes the liquid to form a thin, uniform film over the surface of the plastic wrap due to the adhesive forces that are acting on the liquid (45). However, this very thin film of virus over the plastic wrap allows an increased surface area to volume ratio, resulting in a higher rate of evaporation and desiccation, thus more rapidly reducing viral viability compared to the other materials (Table 2). Our results suggest that plastic wrap can inhibit MHV infectivity over a period of 3 h at 7°C; therefore, plastic wrap may also be able to inhibit other coronaviruses, such as SARS-CoV-2. Further studies will need to be conducted on SARS-CoV-2 and other coronaviruses to see if this result can be replicated.

**TABLE 2:** Half-life and decay rate of the mean MHV PFU/mL on the different materials tested in this study from the initial inoculum (1.0 × 10^4^ PFU/mL)

Sample	Recovered starting PFU/mL	Recovered ending PFU/mL	Time point (h)	Half-life (h)	Decay rate (per h)
Ground beef	1.0 × 10^4^	3.0 × 10^2^	48	9.49	0.07
Stew-cut beef	1.0 × 10^4^	7.0 × 10^2^	48	12.5	0.06
Plastic wrap	1.0 × 10^4^	1.75 × 10^2^	3	0.51	1.35
Meat-absorbent material	1.0 × 10^4^	7.5 × 10^1^	48	6.80	0.10
Styrofoam	1.0 × 10^4^	3.25 × 10^3^	48	29.60	0.02

In contrast to plastic wrap and Styrofoam, when the viral inoculum was added onto the meat-absorbent material, the liquid was readily absorbed. One possible explanation for why the survival of MHV on meat-absorbent material was less than that on Styrofoam could be due to some of the virus being retained in the meat-absorbent materials, reducing the amount collected for the plaque assays; this was evident from the PFU/mL at the early time points, which for meat-absorbent material from 0 to 3 hpi was 8.58 × 10^2^ (± 1.03 × 10^2^), whereas Styrofoam showed a significantly higher viral recovery (2.46 × 10^4^ [± 1.92 × 10^3^] between 0 and 1 hpi and 2.0 × 10^4^ [± 1.63 × 10^3^] at 3 hpi; Table 2). However, being absorbed into the meat-absorbent material could provide a protective barrier for the virus from evaporation. Due to the moist environment and protection of the meat-absorbent material, we hypothesize that this is a possible explanation for why MHV was able to remain infectious for up to 6 hpi before the viability started to decrease.

MHV inoculated on Styrofoam was able to remain viable the longest in comparison to plastic wrap and meat-absorbent material (Table 2). Specifically, we observed a 0.12-log reduction in infectivity for MHV at 3 hpi, and by 48 hpi, we could still detect infectious virus particles on Styrofoam. Overall, the virus incubated on Styrofoam had the smallest decrease in infectivity (0.91-log reduction) compared to plastic wrap and meat-absorbent material (3.12 and 1.11-log reduction, respectively). We propose that this is due to the hydrophobic nature of Styrofoam, which allowed the cohesive forces of the virus in the medium to remain in a large droplet over the 48-h time course, resulting in a lower rate of evaporation and, in turn, increased viral viability.

Our data for MHV demonstrating viability for up to 48 hpi at 7°C on ground beef, stew-cut beef, meat-absorbent material, and Styrofoam are consistent with recent findings of SARS-CoV-2 survival on both meat and other food items stored at 4°C (26–28). Meat processing plants present an ideal environment for the survival and spread of SARS-CoV-2. During processing, the carcass is washed to remove any remaining blood or bone dust and is decontaminated, either through spraying the carcass with high-pressure hot water or through chemical decontamination (46–50). If the carcass is contaminated with SARS-CoV-2 when it is blasted with water, this could potentially allow the virus to become aerosolized and contaminate other carcasses that are being processed, other equipment that is in the vicinity, or the workers in the meat processing plant. After the carcass is washed, it is thoroughly chilled at 4 to 7°C for at least 24 h and cut into larger and smaller chunks of meat, called primal and subprimal cuts (46–50). If there are SARS-CoV-2 particles present on the beef used to produce, for example, ground beef, then the virus could be spread into the meat grinder and contaminate other meat samples that are processed with that equipment (51). Our work used MHV incubated on stew-cut and ground beef, representing subprimal cuts of meat, at 7°C for 48 h to mimic SARS-CoV-2 survival on meat and meat packaging materials as they move through the processing plant (52).

Our findings led us to conclude that MHV, and likely, SARS-CoV-2, can survive on both ground beef and on stew-cut beef for up to 9 hpi before a significant loss in infectivity was detected. In addition, we determined that MHV was still viable to some extent at 48 hpi on both cuts of meat, meat-absorbent material, and Styrofoam. We observed a reduction in MHV infectivity over time for all meat cuts and packaging materials we tested in this study. Coronaviruses rapidly degrade at temperatures above 56°C, so there is little danger from getting infected from SARS-CoV-2 from cooked meat (53, 54). Our results with MHV on ground beef, stew-cut beef, and meat packaging materials suggest that one potential mechanism for SARS-CoV-2 transmission in meat processing plants is from touching meat or packaging materials contaminated with SARS-CoV-2 and then infection via hand-to-face contact (55, 56). However, recent studies on the transfer of SARS-CoV-2 from foods and packaging materials to gloves provide evidence that fomite transmission of SARS-CoV-2 is of minor importance (36, 37). Given the highly infectious nature of SARS-CoV-2, especially variant strains such as Omicron, even relatively low numbers of virus that remain viable for days on food samples or on solid surfaces such as meat-absorbent material or Styrofoam represent a serious health risk, even if the transmission rate of SARS-CoV-2 from surfaces is minimal (36, 37, 57, 58).

Further studies of the different areas of the meat processing plant will allow us to be able to create more accurate models for how SARS-CoV-2 is effectively being spread throughout the meat processing plant. Such models will allow for the development of new methods for the protection of meat processing plant workers from getting infected by SARS-CoV-2 and by new and emerging pathogens. Several strategies that can be employed within meat processing plants are to have policies ensuring workers stay home if they feel sick, encourage regular hand-washing, especially after working in the meat processing plant, rotate the use of different antimicrobial sanitizers for the floors, surfaces, and equipment that is used during meat processing, and have mask-wearing policies in order to help reduce the spread of any illness within meat processing plants.

**Conclusions.** In summary, our results suggest that ground beef, stew-cut beef, meat-absorbent material, and Styrofoam can harbor coronavirus particles (based on MHV used as a surrogate for SARS-CoV-2) which can remain viable for at least 48 h. Furthermore, our study provides evidence that the environmental and physical conditions within meat processing facilities can facilitate the survival of viable virus. Fortunately, recent studies suggest that the transmission rate of SARS-CoV-2 from foods and packaging materials is of minimal importance (36, 37, 57, 58). However, given the highly infectious nature of SARS-CoV-2, the survival of even a modest number of viable virus particles on foods and solid surfaces potentially represents a serious health risk. Repeating this work with SARS-CoV-2 and for extended incubation periods will complete this picture and allow for the implementation of measures to prevent potential COVID-19 health risks from the handling or consumption of meat products.

## MATERIALS AND METHODS

### Cell lines and MHV propagation.

L2 cells (ATCC CCL-149TM) were used for the MHV plaque assay. In addition, the mouse asterocytoma-derived cell line (DBT), was used to propagate MHV (generously provided by Julian Leibowitz, Texas A&M Health Science Center, College Station, TX). All cells used in this study were cultured at 37°C in 5% CO_2_ in Dulbecco’s modified Eagle’s medium (DMEM; Cellgro) supplemented with 10% fetal bovine serum (FBS), penicillin (50 IU/mL), and streptomycin (50 μg/mL). MHV strain A59 (ATCC VR-764) was used for all the experiments in this study. The virus stocks used for this study were produced as previously described (59). The MHV viral titer used for all experiments was ~1.0 × 10^4^ PFU/mL. We chose 100 μL (1.0 × 10^3^ virus particles) to inoculate on each of our samples because 1.0 × 10^2^ – 2.0 × 10^3^ virus particles is the predicted minimal amount of virus particles needed in order to infect someone (60, 61). In addition, we chose 1.0 × 10^3^ virus particles because according to a recent publication modeling the SARS-CoV-2 viral titer when sneezing or coughing on a surface, the range of virus particles for a cough or sneeze was ~1.0 × 10^3^ – 1.0 × 10^5^, which is in this range (62).

### MHV survival on meat.

MHV stocks were cultured to a viral titer of ~1.0 × 10^4^ PFU/mL prior to the start of the experiment. Patties (1 g) of ground beef and stew-cut beef were spot inoculated with 100 μL (1.0 × 10^3^ viral particles in DMEM) of MHV or negative control (100 μL of DMEM [without MHV] was added with 10% FBS onto the meat samples) in a 6-well plate in duplicate (Fig. 1A and B), and each experiment was run twice. All samples were incubated at 7°C for 0, 1, 3, 6, 9, 24, 30, and 48 h. At each time point the virus was harvested, and the stew-cut meat was held with sterile forceps and rinsed with 1 mL of DMEM, which was collected for analysis via RT-qPCR and plaque-assays. For the ground beef samples, the meat was removed with forceps and put into one half of a stomacher bag, with 2 mL of DMEM with 10% FBS and 1% streptomycin/penicillin mix added to the other side. The bag was put into a stomacher and processed for 30 s. Then, the liquid portion was exuded by squeezing and flattening the meat and collected in a sterile cryotube. The recovered MHV supernatant was pipetted up and down multiple times with a 1-mL tip to homogenize the sample.

### MHV survival on meat packaging materials.

MHV stocks were cultured to a viral titer of ~1.0 × 10^4^ PFU/mL prior to the start of the experiment. Plastic wrap, meat-absorbent materials, and Styrofoam were cut into squares (18 by 18 by 2 mm) and were placed in a six-well plate to be spot-inoculated with MHV. The meat packaging materials (plastic wrap, meat-absorbent material, and Styrofoam) were spot inoculated with 100 μL virus (1.0 × 10^3^ viral particles in DMEM) or control (DMEM). The plastic wrap used was made of polyethylene, which is the same type used in retail meat packaging (63, 64). The meat-absorbent material was made of a silica gel and a polyethylene plastic wrap (65, 66). The experiments were set up in duplicate in a 6-well plate; each experiment was performed twice (Fig. 1C to E). To simulate the conditions of the meat processing plant, the meat packaging materials and virus were incubated at 7°C for 0, 1, 3, 6, 9, 24, 30, and 48 h in duplicate. Meat processing plants are kept between 4 and 7°C, so we chose 7°C, at the upper range of temperatures found inside meat processing plants, since previous literature has shown that SARS-CoV-2 can survive at 4°C (26–28). We chose to incubate the virus for 48 h as a proof of concept that the virus can remain viable for up to 2 days. For the negative control, 100 μL of DMEM was added (without virus) with 10% FBS onto the meat packaging materials. At each time point, the meat packaging materials were held with sterile forceps and were rinsed with 1,000 μL of DMEM with 10% FBS. For the meat-absorbent materials, the virus was squeezed out of materials using the forceps. The samples were collected in a sterile cryotube. The homogenate was mixed several times by pipetting up and down. The homogenate was used for RT-qPCR and plaque assay analysis. Results from this experiment are the mean values and standard deviations (error bars) from two independent experiments, with technical replicates completed for each sample in each experiment. Statistical analyses were completed using GraphPad Prism, and an independent *t* test was completed to determine the *P* value and significance of each sample.

### Viral RNA extraction.

Viral RNA was extracted and purified for each sample using the Monarch total RNA miniprep kit from New England Biolabs (NEB) following the Tough-to-Lyse protocol. Then, 400 μL of 1× DNA/RNA protection reagent was added to 400 μL of harvested sample and placed in a water bath sonicator for 10 min and then vortexed for 1 min. The sonication/vortex step was repeated, and the lysate was centrifuged at 16,000 × *g* for 2 min. Next, 800 μL of the supernatant was applied to a spin column and centrifuged, the pass-through was discarded, and the column washed two times with 500 μL of wash buffer (as per the manufacturer’s protocol). Purified RNA was eluted from the column using 100 μL of molecular-grade, RNase/DNase-free water. Purified RNA samples were quantified using a NanoDrop instrument. RNA samples were stored at −20°C until ready for RT-qPCR analysis. Results from this experiment are the mean values and standard deviations (error bars) from two independent experiments, with technical replicates completed for each sample in each experiment. Statistical analyses were completed using GraphPad Prism, and an independent *t* test was completed to determine the *P* value and significance of each sample.

### MHV RT-qPCR analysis.

RT-qPCR analysis was completed using NEB’s Luna universal probe one-step RT-qPCR kit. Purified RNA extracted from MHV was used for the positive control and standard curve. The RT-qPCR analyses were completed in 25-μL reactions, containing 10 μL of Luna universal probe one-step reaction mix, 1 μL of Luna WarmStart RT enzyme mix, 400 nM forward primer (5′-GGAACTTCTCGTTGGGCATTATACT-3′), 400 nM reverse primer (5′-ACCACAAGATTATCATTTTCACAACATA-3′), 200 nM probe (5′-FAM-ACATGCTAC-ZEN-GGCTCGTGTAACCGAACTGT-3′IABkFQ-3′), 250 ng RNA, and nuclease-free water (67). The RT-qPCR analysis was performed using a Bio-Rad CFX96 deep-well real-time thermal cycler. Thermal cycling conditions were 55°C for reverse transcription for 10 min, denaturation and polymerase activation at 95°C for 1 min, and 40 cycles of 95°C for 15 s followed by 60°C for 30 s for data collection. RT-qPCRs were performed in quadruplicate for each sample, and the quantification cycle (Cq) was used for data analysis. Gene copy numbers were calculated by comparing the Cq value for 250 ng MHV RNA on the standard curve with the Cq value for each sample. The following equation was used to calculate the gene copy numbers for the M gene of MHV: gene copy number = (copy number of 250 ng of positive control) – ([Cq pos. cont. – Cq exp. cont.]/Cq exp. cont.) × (copy number of 250 ng of positive control) (Cq pos.cont., quantification cycle positive control concentration; Cq exp. cont., quantification cycle experimental sample concentration) (68). Data from each sample were compared using positive and negative controls performed in duplicate. Results from this experiment are the mean values and standard deviations (error bars) from two independent experiments, with technical replicates completed for each sample in each experiment (Fig. S1). Statistical analyses were completed using GraphPad Prism, and an independent *t* test was completed to determine the *P* value and significance of each sample.

### Plaque assay analysis.

Plaque assays were performed to determine MHV infectivity/viability. Each sample was filtered through a 0.2-μm syringe filter to remove bacterial contaminants before being serially diluted in DMEM with 2% FBS and 1% streptomycin/penicillin mix. Then, 100 μL was used to infect 1.0 × 10^6^ L2 cells seeded in a six-well plate, in duplicate. A previously published protocol was followed for the solid double-layer plaque assay (69). The percent recovery was calculated by dividing the PFU number at time zero (*T*_0_) by the original PFU number of the viral stock. The fold change was calculated in Table 1 by dividing the PFU number at *T*_0_ and the PFU number at a given time point. The half-life and decay rate values were calculated using the half-life formulas shown here: *N*(*t*) = *N*_0_(1/2)*^t^*^/^*^t^*_1/2,_ *N*(*t*) = *N*_0_*e*^–^*^t^*^/τ^, and *N*(*t*) = *N*_0_*e*^–λ^*^t^*, where *N*_0_ is the initial quantity, *N*(*t*) is the remaining quantity after time, *t*, *t*_1/2_ is the half-life, τ is the mean lifetime, and λ is the decay constant. Results from this experiment are the mean values and standard deviations (error bars) from two independent experiments, with technical replicates completed for each sample in each experiment (Fig. 1).
