# Supplementary material for: Murine Hepatitis Virus, a Biosafety Level 2 Model for SARS-CoV-2, Can Remain Viable on Meat and Meat Packaging Materials for at Least 48 Hours
Source: Microbiol Spectr. 2022 Sep 7;10(5):e01862-22. doi: 10.1128/spectrum.01862-22 (PMC9603800; doi:10.1128/spectrum.01862-22)
Supplement: Supplemental file 1 — Supplemental material. Download spectrum.01862-22-s0001.pdf, PDF file, 0.1 MB [file spectrum.01862-22-s0001.pdf]

Supplementary Results & Discussion

Viral stability as determined by RT-qPCR.

When comparing survival on two different cuts of meat, we observed via RT-qPCR that viral RNA was able to remain stable for up to 1 hpi on the ground beef before a ~6% decrease in gene copies was detected at 3 hpi (Supplemental Fig. 1A, Table S1). However, plaque assay data suggested that viral viability remained constant from one to six hpi. The viral RNA was observed to continue to degrade at each timepoint by both testing methods, except at 9 hpi for RT-qPCR, which we consider an anomaly and not significant to our data, as it was not replicated in terms of PFU/mL (Supplemental Fig. 1A and Fig. 1A, Table S1). By 48 hpi, a 16.5% reduction in viral RNA was identified, and a similar reduction was observed via plaque assay at timepoint 9 hpi (Supplemental Fig. 1A and Fig. 1A, Table S1). This suggests that the virus particles can remain stable on ground beef for up to 6 hpi before starting to destabilize at 9 hpi.

For stew-cut beef, the MHV M-gene copy numbers started at 6.76 gene copies/µL at 0 hpi (Supplemental Fig. 1B, Table S1). We observed a significant decrease in the gene copy numbers for the M-gene of MHV at 9 hpi (a 15.4% reduction from its starting value) (Supplemental Fig. 1B, Table S1). At 24 and 30 hpi, we did not detect a significant decrease in the M-gene copies compared to the starting value at 0 hpi, although we are unable to identify what is significant about these times, as by 48 hpi we observed a 6.3% reduction compared to 0 hpi (Supplemental Fig. 1B, Table S1). The variability in the qPCR results was most likely due to several factors. 60% of the virus sample was absorbed into the ground beef and 79.7% of the virus sample was absorbed into the stew-cut beef; therefore, a large proportion of the virus was lost/not collected for both cuts. Also, the MHV-on-meat samples were incubated in a refrigerated and fan-driven incubator, suggesting a percentage of the inoculation droplet may have

evaporated during the test period. Lastly, there may have been a percentage of the virus that was not collected when the meat samples were washed, with a small percentage of the viral supernatant being left in the bottom of each well.

When testing the ability of MHV to survive on packaging materials by RT-qPCR we detected a significant decrease in copy numbers on plastic wrap after 9 hpi (Supplemental Fig. 1C, Table S1). However, we saw no significant decrease in the MHV M-gene copy numbers on the meat-absorbent material, or on Styrofoam from 0 to 48 hpi, despite differences detected in the infectivity of the virus (demonstrated by plaque assay) on these materials (Supplemental Fig. 1C-E and Fig. 1C-E, Table S1). This suggests that the virus particles had started to lose viability after 9 hpi, but as RT-qPCR analysis will still quantitate vRNA from non-viable virial particles there was no change in Cq values over time for the meat-absorbent materials and for the Styrofoam samples.

Supplementary Fig. S1.

qPCR Analysis of MHV collected from ground beef, stew-cut beef, plastic wrap, meat-absorbent material, and Styrofoam samples post-incubation. RT-qPCR analysis of the MHV M-gene following 1 x 10^4 PFU/mL inoculated on (A) ground beef, (B) stew-cut beef, (C) plastic wrap, (D) meat-absorbent material, or (E) Styrofoam. Results in this figure are the mean values from quadruplicate reactions, and standard deviations (error bars) from two independent experiments, with technical duplicates for each sample in each experiment. Statistical significance was analyzed by unpaired t-test. ns: not significant; *: $p < 0.05$; **: $p < 0.01$; ***: $p < 0.001$; ****: $p < 0.0001$.

Supplementary Figure 1.

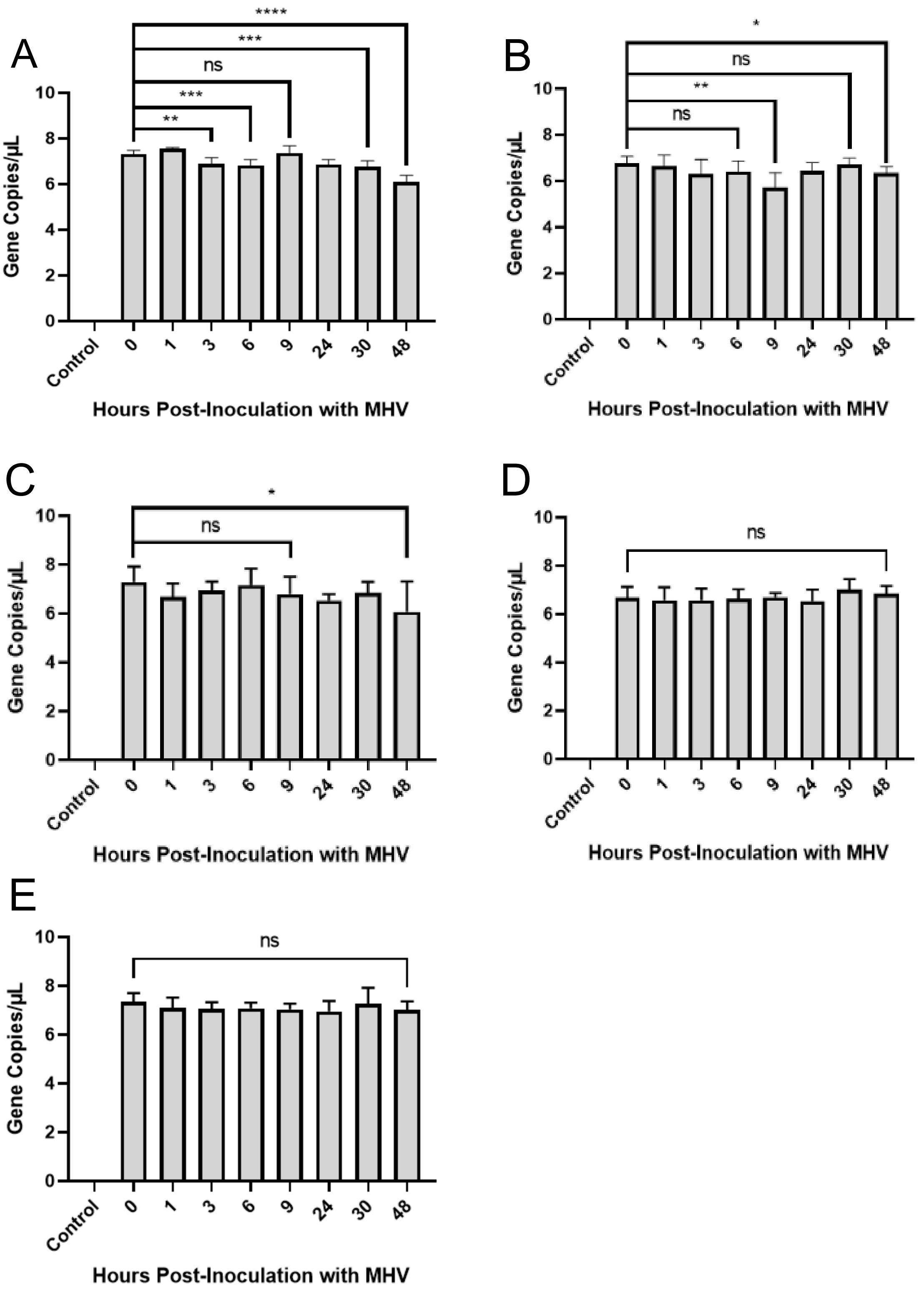

Supplementary Table S1: Data from the RT-qPCR analysis on the recovered MHV RNA from the different experimental conditions, Cq numbers, and the percentage and fold change from the initial inoculum (1.0 x 10^4 PFU/mL).

**Sample**	**M gene Cq @ 0 hpi**	**M gene Cq @ 1 hpi (% change/fold change)**	**M gene Cq @ 3 hpi (% change/fold change)**	**M gene Cq @ 6 hpi (% change/fold change)**	**M gene Cq @ 9 hpi (% change/fold change)**	**M gene Cq @ 24 hpi (% change/fold change)**	**M gene Cq @ 30 hpi (% change/fold change)**	**M gene Cq @ 48 hpi (% change/fold change)**
Ground beef	15.11 ±0.25; 14.50 ±0.07.	(+6.2%, 1.06-fold); (+1.2%, 1.01-fold).	(-1.5%, 1.02-fold); (-11.7%, 1.12-fold).	(-2.5%, 1.03-fold); (-12.4%, 1.12-fold).	(-0.05%, 1.01-fold); (+1.9%, 1.02-fold).	(-4.4%, 1.04-fold); (-9.4%, 1.09-fold).	(-9.2%, 1.09-fold); (-7.5%, 1.08-fold).	(-15.2%, 1.15-fold); (-21.4%, 1.21-fold).
Stew-cut beef	17.43 ±1.10; 17.29 ±0.14.	(+3.9%, 1.04-fold); (-7.3%, 1.07-fold).	(-4.2%, 1.04-fold); (-8.6%, 1.09-fold).	(+0.0%, 1.00-fold); (-10.2%, 1.10-fold).	(-15.5%, 1.15-fold); (-13.2%, 1.13-fold	(+0.0%, 1.00-fold); (-9.3%, 1.09-fold).	(+2.1%, 1.02-fold); (-3.3%, 1.03-fold).	(-4.5%, 1.04-fold); (-7.2%, 1.07-fold).
Plastic wrap	15.49 ±0.31; 22.04 ±0.20.	(-12.8%, 1.13-fold); (-5.6%, 1.06-fold).	(-9.8%, 1.10-fold); (-1.8%, 1.02-fold).	(-1.8%, 1.02-fold); (-1.8%, 1.02-fold).	(-7.6%, 1.08-fold); (-7.4%, 1.07-fold).	(-18.3%, 1.18-fold); (-5.4%, 1.05-fold).	(-12.7%, 1.13-fold); (-1.3%, 1.01-fold).	(-15.9%, 1.16-fold); (-19.8%, 1.2-fold).
Meat-absorbent material	21.12 ±1.55; 21.05 ±0.91.	(+1.1%, 1.01-fold); (-4.0%, 1.04-fold).	(+1.5%, 1.02-fold); (-4.4%, 1.04-fold).	(-1.6%, 1.02-fold); (+0.7%, 1.01-fold).	(-3.5%, 1.04-fold); (+3.8%, 1.04-fold).	(+0.7%, 1.07-fold); (-4.4%, 1.04-fold).	(+5.3%, 1.06-fold); (+4.1%, 1.04-fold).	(+3.4%, 1.04-fold); (+1.0%, 1.01-fold).
Styrofoam	15.01 ±1.19; 15.44 0.32.	(-0.7%, 1.01-fold); (-6.4%, 1.06-fold).	(-2.2%, 1.02-fold); (-6.5%, 1.07-fold).	(-2.3%, 1.02-fold); (-5.9%, 1.06-fold).	(-2.9%, 1.03-fold); (-6.4%, 1.06-fold).	(-1.5%, 1.02-fold); (-10.0%, 1.1-fold).	(+6.7%, 1.07-fold); (-8.7%, 1.09-fold).	(-1.7%, 1.02-fold); (-8.0%, 1.08-fold).
